# Phosphate-solubilizing Bacteria from Safflower Rhizosphere and their Effect on Seedling Growth

**DOI:** 10.1515/biol-2019-0028

**Published:** 2019-07-10

**Authors:** Tingting Zhang, Feng Hu, Lei Ma

**Affiliations:** 1 College of Resources and Environmental Sciences, Nanjing Agricultural University, Nanjing, Jiangsu, 210095, China

**Keywords:** *Acinetobacter* spp, *Sinorhizobium* spp, Plant-growth promotion, Rhizobacteria, Co-inoculation

## Abstract

Phosphate-solubilizing bacteria (PSB) can convert insoluble rhizosphere phosphorus into forms that are absorbable by plants and thus enhance the growth of plants. Safflower is a cash crop that is a source of vegetable oils, food coloring and flavoring agents. This study sought to isolate PSB in safflower rhizosphere soil and investigate their effects on seedling growth. The isolated PSB were identified as belonging to the genera *Pseudomonas*, *Sinorhizobium*, *Staphylococcus*, *Acinetobacter* and *Enterobacter* using 16S rRNA gene sequence analysis. *Acinetobacter* sp RC04. showed the best performance in phosphate solubilization, with the efficiency of the process being influenced by carbon source, nitrogen source, cultivation temperature and initial culture pH. *Acinetobacter* sp. RC04 and *Sinorhizobium* sp. RC02 showed the ability to improve safflower seed germination and, when co-inoculated, improved seedling growth. Hence, we suggest that *Acinetobacter* sp. RC04 and *Sinorhizobium* sp. RC02 could be developed for field application to promote safflower growth. The results from this study will help drive novel biofertilizer discovery and could be included in integrated nutrient management regimes for safflower and other important economic crops.

## 1 Introduction

Phosphorus (P) is an essential nutrient for crop growth and development [1, 2, 3]. In the soils of some agroecosystems, such as some arid and semi-arid regions, the total soil P concentration is adequate, but the content of soluble or plant-absorbable P (HPO_4_^2−^ or H_2_PO_4_^−^) is deficient [4,5]. In these soils, P tends to be limited because of immobilization and precipitation. For example, P is fixed by free oxides and hydroxides of aluminium and iron in acid soils and by calcium in alkaline soils, resulting in the low efficiency of soluble P fertilizers [6]. Solubilization of mineral phosphorus is beneficial to enhance plant growth.

Phosphate-solubilizing bacteria (PSB), associated with the plant rhizosphere, make mineral phosphorus more readily available for plant uptake by transforming insoluble P into forms that are available for the crop [7]. The effect of PSB is considered a mechanism for enhancing plant growth. Until now, previously known PSB belong to numerous genera including *Pseudomonas*, *Bacillus*, *Rhizobium*, *Burkholderia*, *Achromobacter*, *Agrobacterium*, *Microccocus*, *Aereobacter*, *Flavobacterium* and *Erwinia* [4]. Inoculation with these phosphate dissolvers as biofertilizers has been reported to increase P uptake and promote plant growth [8, 9, 10]. For example, treatment with PSB has increased the yield of wheat and promoted the growth of rice [11,12]. However, the ability of PSB to solubilize phosphate varies by bacterial species. The long-term stability and capability of phosphate dissolvers are key problems for their widespread application in promoting crop yields [13,14].

Safflower (*Carthamus tinctorious* L.) is a cash crop; its seeds are used for extraction of vegetable oil, and its petals are dried and used as food coloring and flavoring [15]. Safflower is suitable for cultivation in arid and semiarid regions in the Far East, central and northern Asia and the European Caucasus regions because of its drought tolerance and salt resistance [16]. As an important industrial and multipurpose crop, the global production of safflower exceeds 600 million tons per year [15]. However, PSB strains in the safflower rhizosphere still await investigation.

The present study aimed to isolate plant-growth-promoting PSB from safflower rhizosphere soil. Among those isolates, *Acinetobacter* sp RC04. showed the best performance in phosphate solubilization. To better understand its role in the rhizosphere, this study then surveyed the potential of the *Acinetobacter* sp RC04. It showed the ability to improve safflower seed germination and, when co-inoculated with *Sinorhizobium* sp. RC02, improve seedling growth. The results from this study could help drive novel biofertilizer discovery for safflower and other crops.

## 2 Materials and methods

### 2.1 Soil sample collection

This study was carried out at the agricultural experiment station of Shihezi University in Shihezi City, Xinjiang Province, China (44.27° N, 85.94° E), using the safflower cultivar Xinhong 4 as the model. The soil of the sampling sites is heavy loam with pH 8.0. According to the records of the station, the soil layer is approximately 15–21 cm thick and contains approximately 3.2 t ha^−1^ total P with 54 kg ha^−1^ available P, 1.6 t ha^−1^ total nitrogen with 88 kg ha^−1^ available nitrogen, 52 t ha^−1^ total potassium with 366 kg ha^−1^ available potassium and 30 t ha^−1^ soil organic matter. Samples were collected from soil sites not exposed to agrochemicals for several years. Sampling spots were selected by following an S pattern in the field. Rhizosphere samples (including roots and soil adhering to the roots) were collected at depths of 0–15 cm. Three biological replicates of plants were obtained at each spot. The samples were placed individually in sterile plastic bags and stored immediately in a cooler until arrival at the laboratory. All samples were stored at 4 °C until analysis and isolation.

### 2.2 PSB isolation

Excess soil was shaken from roots, leaving approximately 1 mm of soil still attached to the roots. About 1 g of the soil tightly adhering to the roots was separated from the roots by shaking in a sterile flask containing 50 ml of sterile phosphate-buffered saline (PBS) solution. The suspension was centrifuged twice for 1 min at 12,000 × *g*, serially diluted in PBS solution (10^−2^, 10^−4^, and 10^−6^) and plated on the P solubilization medium described below.

The P solubilization medium was modified from the National Botanical Research Institute’s phosphate-growth medium [17]. Tricalcium phosphate (TCP) was the sole P source. The medium contained (per liter) 10 g of glucose, 5 g of Ca_3_(PO_4_)_2_, 5 g of MgCl_2_·6H_2_O, 0.25 g of MgSO_4_·7H_2_O, 0.2 g of KCl, 0.5 g of (NH_4_)_2_SO_4_, 0.3 g of NaCl, 0.03 g of FeSO_4_·7H_2_O and 0.4 g of yeast extract in distilled water. Agar (20 g) was added to the medium for plate assays. The medium pH was adjusted to 7.0–7.5.

After 6 days of incubation on the P solubilization medium plates at 30 °C, colonies surrounded by a clear halo were considered phosphate dissolvers. Colonies were purified by restreaking on a plate. The capacity to dissolve phosphate on solid medium was measured, after 6 days of incubation at 30°C, as the ratio between the diameter of the phosphate solubilization halo around the colony and the diameter of the colony itself. The experiment was performed on a total of 9 plates, accounting for a total of 50 colonies.

### 2.3 Quantitative estimation of P solubilization in liquid culture

The potential PSB were grown in 100 mL of P solubilization medium and incubated with shaking at 300 rpm at 30 °C for 24 h. The approximate number of colony-forming units per milliliter (CFU mL^−1^) was determined by optical density measurement and serial dilutions with plate counts. One milliliter of culture (10^6^ CFU mL^−1^) was transferred to a 500-mL Erlenmeyer flask containing 100 mL of P solubilization medium and incubated on a gyratory shaker (200 rpm) at 30 °C. The soluble P and optical density at 600 nm were measured every 12 h for 120 h using the phosphomolybdate blue colorimetric method [17]. Experiments were conducted five times per isolate. After the predefined incubation period, the cultures were harvested by centrifugation at 3000 × *g* for 15 min and the supernatant was filtered with 0.45-μm syringe filters for analysis of P concentration in the medium. Sterile, non-inoculated medium served as the control.

### 2.4 P solubilization assays

First, one-factor-at-a-time experiments were used to estimate whether a factor in PSB liquid cultivation had any effect on P solubilization and to seek to optimize the response. Carbon source, nitrogen source, initial pH, initial inoculum and cultivation temperature were separately tested. Each experimental factor was tested to determine optimum, keeping all other experimental factors constant as described above. After determination of the optimum of a given factor, the factor was subsequently held at the optimum throughout the remaining trials.

The carbon sources (20 g L^−1^) in liquid culture tested were fructose, glucose, sorbitol, sucrose and soluble starch. The nitrogen sources tested were (1.5 g L^−1^) (NH_4_)_2_SO_4_, NH_4_Cl, NH_4_NO_3_, urea and beef extract. Initial pH was 4, 5, 6, 7 or 8. For the inoculum assay, the initial bacterial cell suspension concentration was 3×104, 4×104, 5×104, 6×10^4^ or 7×10^4^ CFU mL^−1^. The cultivation temperature was 20, 25, 30, 35 or 40 °C. Each treatment had five replicates. After 6 days of cultivation, the bacterial cell suspension concentration was adjusted to 10^8^ CFU mL^−1^ to estimate P solubilization.

To test possible factorial interactions influencing P solubilization, a full factorial experiment was performed. With five factors each taking two levels, the experiment had 32 treatment combinations (each having three replicates). It tested the effects of the five independent variables (NH_4_Cl concentration, glucose concentration, cultivation temperature, initial pH and inoculum amount) on the dependent variable (P solubilization) and possible interactions. The levels of the variables were designed according to the preceding one-factor-at-a-time experiments: [NH_4_Cl] had levels 1 g L^−1^ and 3 g L^−1^, [glucose] had levels 10 g L^−1^ and 30 g L^−1^, the temperature was 25 °C or 35 °C, the initial pH was 5.5 or 6.5 and the initial bacterial cell suspension concentration was 4×10^4^ or 6×10^4^ CFU mL^−1^. All other assay conditions were as described above.

### 2.5 PSB isolates identification

Bacterial genomic DNA was extracted by the phenol/chloroform method [18]. The 16S ribosomal DNA (rRNA) was amplified from extracted DNA by polymerase chain reaction with primers 5′-AGAGTTTGATCCTGGCTCAG-3′ and 5′-ACGGTTACCTTGTTACGACTT-3′ [19]. Amplification was performed by initial denaturation at 95 °C for 3 min, followed by 35 cycles of 95 °C for 30 s, 51 °C for 30 s and 72 °C for 1 min, with a final extension at 72 °C for 10 min. The 50-μL PCR mixtures contained 0.2 μM of each primer, 0.2 mM dNTPs, EasyTaq® buffer, 2.5 U EasyTaq® DNA polymerase (TRANSGEN, China) and 10 ng of template DNA. The PCR products were sequenced by Beijing Sun Biotech Co., Ltd. (Beijing, China). The sequences were then aligned to reference 16S rRNA sequences in the NCBI database using the BLAST program with default parameters [20]. The sequenced 16S rRNAs and reference 16S rRNAs were subjected to phylogenetic analysis using MEGA7 software [21]. The sequences were aligned by ClustalW to reconstruct a phylogenetic tree using the Kimura 2-parameter distance model and neighbor-joining method (1000 bootstrap replicates).

### 2.6 Germination assays

One milliliter of 24-h-old bacterial cultures was inoculated into 100 mL of Luria–Bertani (LB) medium (10 g L^−1^ tryptone, 5 g L^−1^ yeast extract and 10 g L^−1^ NaCl at pH 7.0), shaken for 72 h at 120 rpm at 30°C, and centrifuged for 10 min at 9,400 × *g*. The supernatant was discarded, and the pellet was resuspended in distilled water. Safflower seeds were surface sterilized with 5% sodium hypochlorite (commercial laundry bleach) for 15 min, and rinsed five times with sterile water.

The seed germination assay was based on a completely randomized design with three PSB inoculation treatments: (1) control without bacterial inoculation; (2) inoculation of one PSB strain (10^3^ CFU mL^−1^); and (3) co-inoculation of two PSB strains (each with 10^3^ CFU mL^−1^). Sterilized safflower seeds were placed on a filter paper in a plate (9 cm diameter). A five milliliter suspension of one treatment was added to one plate. Each treatment included at least three biological replicates. The seeds were germinated for 3 days at 28 °C in the dark.

### 2.7 Effects of PSB on plant growth

Sterilized seeds were sown in plastic pots (1 L) filled with autoclaved (121°C for 60 min) loamy soil and sand in 1:1 ratio (v/v) at 25 °C in a plant growth chamber (16-h light and 8-h dark, 40 ± 10 % relative humidity). Plants were watered with an equal volume of autoclaved sterilized water to keep the soil moist when needed. A pot with one seedling was considered an experimental unit, and three replicates per treatment were set up in a completely randomized design.

The PSB inoculation treatment for the greenhouse pot assay was designed with three treatments: (1) control without bacterial inoculation; (2) inoculation of one PSB strain (10^6^ CFU mL^−1^); and (3) co-inoculation of two PSB strains (each with 10^6^ CFU mL^−1^). Each treatment had three pots. A five milliliter suspension was inoculated on the top of the seed and the soil nearby at the time of planting. After 5 days, another 5 mL of suspension was added to the soil around the seeding area. Distilled water was used as a control. No other nutrients or bacterial inocula were supplied. Seedlings were harvested 4 weeks after sowing.

### 2.8 Data analysis

Differences were tested using ANOVA and groups were tested using Tukey’s HSD multiple comparisons procedure. Effects of factors in the P solubilization assay were evaluated using a GLM procedure (Gaussian error distribution).

**Ethical approval**: The conducted research is not related to either human or animals use.

## 3 Results and discussion

### 3.1 Characterization of PSB

**Figure 1:**
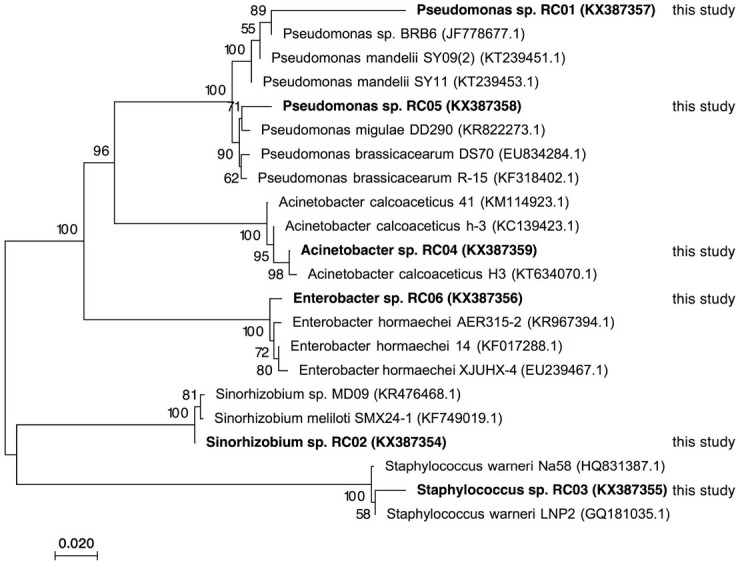
Evolutionary relationships of the phosphate-solubilizing bacteria (PSB) isolated in the present study and related strains in the NCBI database based on 16S rRNA gene sequences. The percentages of replicate trees in which the associated taxa clustered together in the bootstrap test (1000 replicates) are shown next to the branches. The tree is drawn to scale with branch lengths similar to those of the evolutionary distances used to infer the phylogenetic tree. Bacterial strains in boldface indicate PSB included in the present study. Accession numbers are provided in parentheses.

On screening isolates from the safflower rhizosphere, six PSB strains were identified by their production of a halo around colonies on plates containing P solubilization medium. The 16S rRNA gene sequences of these strains were determined and deposited in the NCBI nucleotide sequence database (Table 1). They were clustered into the genera *Pseudomonas*, *Sinorhizobium*, *Staphylococcus*, *Acinetobacter* and *Enterobacter* by phylogenetic analysis (Figure 1). Their biochemical and physiological characteristics are shown in Table 1.

**Table 1:** Characteristics of PSB.

Strain	NCBI Accession	*D*/*d*	P (mg L^−1^)	Oxygen	Oxidase	Catalase	Starch	Methyl-Red	Indole Reduction
*Pseudomonas* sp.	KX387357	3.17±0.13bc	131.6±1.79c	+	+	+	+	+	+
RC01									
*Sinorhizobium* sp.	KX387354	2.32±0.18d	90.9±1.56e	+	+	+	−	+	−
RC02									
*Staphylococcus* sp.	KX387355	2.58±0.16cd	112.5±1.91d	+	−	+	−	+	−
RC03									
*Acinetobacter* sp.	KX387359	4.08±0.13a	168.5±1.27a	+	+	+	−	−	+
RC04									
*Pseudomonas* sp.	KX387358	3.33±0.12b	157.2±1.21b	+	+	+	−	+	+
RC05									
*Enterobacter* sp.	KX387356	2.35±0.19d	92.4±0.75e	+	−	+	−	−	−
RC06									

Six PSB strains were identified and their 16S rRNA gene sequences were deposited in the NCBI nucleotide sequence database. *D*, diameter of phosphate solubilization circle around the colony; *d*, diameter of the colony. P, phosphate solubilization. Values are the mean and standard error (*n* = 5). Means marked with different letters were significantly different (TukeyHSD, *P* < 0.05). The results of oxygen utilization, oxidase test, catalase test, starch utilization, methyl red test and indole reduction were given. +, tested positive or used as substrate; −, tested negative or not used as substrate.

The halo ratios of the six strains ranged between 2.32 and 4.08 after 6 days of incubation. The concentrations of soluble P they produced ranged between 90.9 and 168.5 mg L^−1^ in P solubilization medium. The strain *Acinetobacter* sp. RC04 showed the highest P solubilization among the strains (ANOVA, *F*_(5, 24)_ = 504.4, *P* < 10^−15^; TukeyHSD, *P* < 0.05).

### 3.2 Growth profile of, and phosphate solubilization by, *Acinetobacter* sp. RC04

The growth profile of *Acinetobacter* sp. RC04 (judged by OD_600_) consisted of lag (0–12 h), exponential (12–36 h) and plateau (after 36 h) phases (Supplementary figure). The biomass (OD_600_) reached its maximum (1.25–1.29) at 48 h. The capacity to dissolve TCP also reached its maximum (182.77–184.31 mg L^−1^) at 48 h. The growth profile showed a similar trend to the phosphate solubility (Spearman’s rank correlation *rho* = 0.95, *P* < 10^−16^).

### 3.3 Effect of cultivation conditions on P solubilization by *Acinetobacter* sp. RC04

**Figure 2:**
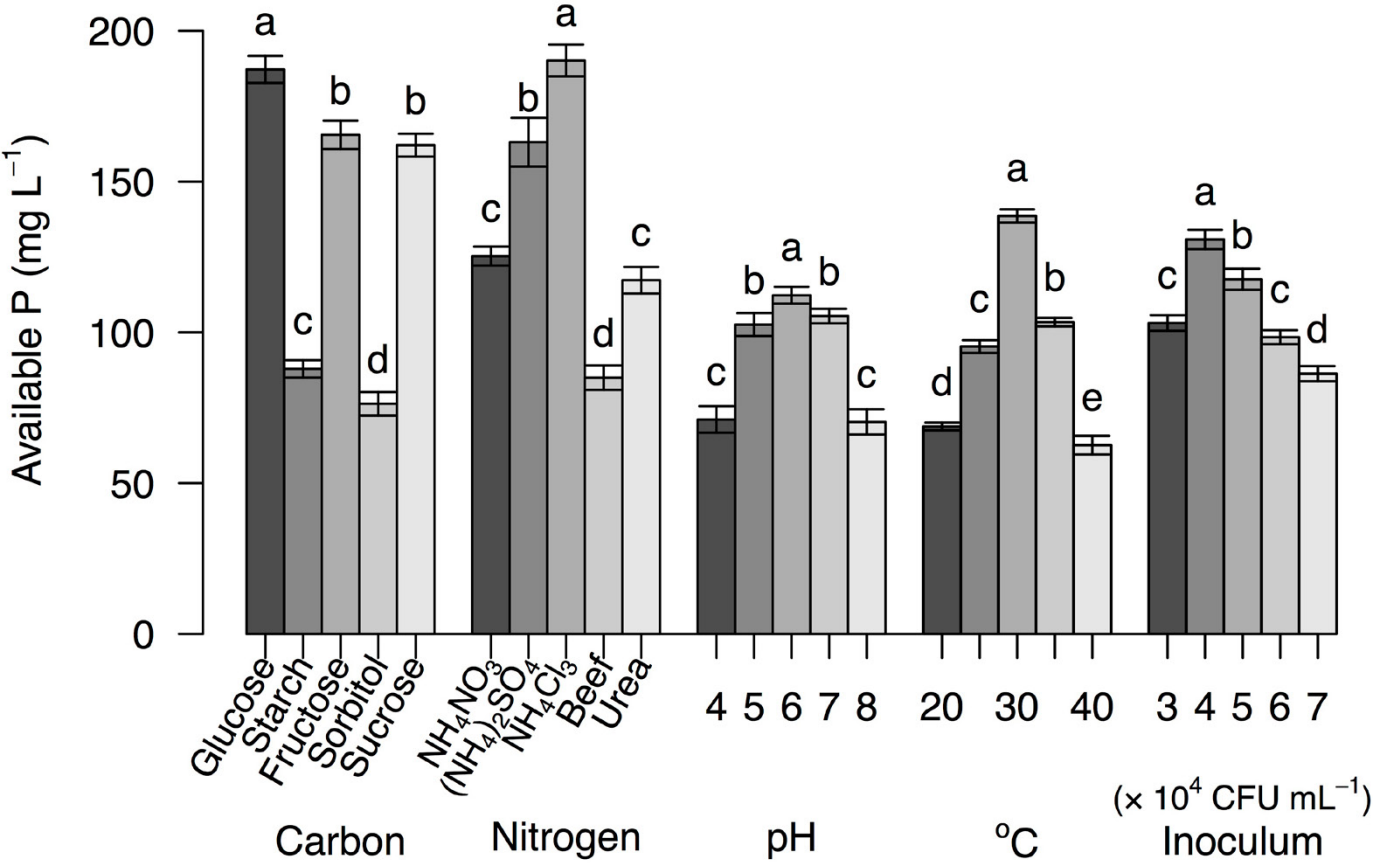
P solubilization assays of *Acinetobacter* sp. RC04. Bars represent means, and error bars show standard errors (*n* = 5). The means within one group marked with different lowercase letters were significantly different at *P* < 0.05 (TukeyHSD).

The cultivation conditions had a significant effect on the ability of *Acinetobacter* sp. RC04 to solubilize phosphate (Figure 2). The available P reached the maximum values with glucose as the carbon source, NH_4_Cl as the nitrogen source, initial pH = 6.0, initial inoculum of 4×10^4^ CFU mL^−1^, and cultivation at 30 °C (Figure 2). Table 2 shows the effect of cultivation conditions ([NH_4_Cl], [glucose], cultivation temperature, initial pH and inoculum amount) on P solubilization and possible interactions between these factors. Interactions were observed between [glucose] and temperature, between [glucose] and [NH_4_Cl], and between pH and temperature (*P* < 0.001, GLM).

**Table 2:** Generalized linear model.

Terms	Generalized linear model				
		Coefficients	*t* value	Pr (>|*t*|)	Sign
(Intercept)		1499.94	7.08	5×10^−10^	***
NH_4_Cl (g L^−1^)		−77.00	−2.69	9×10^−03^	**
Glucose (g L^−1^)		11.40	3.99	1×10^−04^	***
pH		−183.22	−5.55	4×10^−07^	***
Temp. (°C)		−28.59	−5.43	6×10^−07^	***
Inoculum (× 10^4^ CFU mL	^−1^)	−102.24	−3.77	3×10^−04^	***
NH_4_Cl : Glucose		−0.80	−4.07	1×10^-04^	***
NH_4_Cl : pH		2.27	0.58	0.57	
NH_4_Cl : Temp.		1.00	2.53	0.01	*
NH_4_Cl : Inoculum		4.09	2.07	0.04	*
Glucose : pH		−0.71	−1.79	0.08	
Glucose : Temp.		−0.15	−3.92	2×10^−04^	***
Glucose : Inoculum		0.24	1.21	0.23	
pH : Temp.		3.76	4.77	8×10^−06^	***
pH : Inoculum		8.09	2.05	0.04	*
Temp. : Inoculum		0.71	1.81	0.07	
Residuals					

Generalized linear model was used to test the effects of the five independent variables (NH_4_Cl concentration, glucose concentration, initial pH, cultivation temperature and initial inoculum amount) on the response variable (P solubilization) and possible interactions. Sign, ‘***’ Pr < 0.001; ‘**’< 0.01; ‘*’< 0.05.

Compared with other carbon sources, glucose significantly promoted the P solubilizing capacity of *Acinetobacter* sp. RC04 (ANOVA, *F*_(4, 20)_ = 468.1, *P* < 10^−15^, Figure 2). In addition, the amount of glucose played a role in P solubilization according to the GLM (Table 2). These results were consistent with the report that glucose could induce catabolite repression and affect the activity of acid and alkaline phosphatases in PSB [22]. Among the different nitrogen sources tested, NH_4_Cl was the best for *Acinetobacter* sp. RC04 to solubilize P (ANOVA, *F*_(4, 20)_ = 243.8, *P* < 10^−15^). This result supports a previous suggestion that NH_4_Cl could be used as a nitrogen source to promote growth of PSB [17]. Moreover, a significantly negative interaction existed between NH_4_Cl and glucose concentrations (Table 2). The optimum P solubilization was obtained with the highest glucose concentration and the lowest NH_4_Cl concentration, consistent with previous reports that carbon and nitrogen concentrations modulate P solubilization efficiency [23, 24, 25].

Temperature influenced the efficiency of P solubilization by *Acinetobacter* sp. RC04 (ANOVA, *F*_(4, 20)_ = 893.7, *P* < 10^−15^). Temperature plays a crucial role in influencing the activity of phytases [26]; the enzyme phytase releases P from phytate. Figure 2 shows that the P concentration increased with increasing temperature up to 30 °C, then decreased at higher temperatures. The trend is similar to that for *Aspergillus oryzae* and *A. niger* [26], although the optimal temperatures were different. The temperature of the highest activity of phytase varies widely for different microorganisms [27, 28, 29, 30, 31, 32]. In addition, pH also influenced the P solubilization (ANOVA, *F*_(4, 20)_ = 169.4, *P* < 10^−14^). The P solubility was highest at pH 5–7 and lower at pH 4 and 8.

### 3.4 PSB effect on seedling growth

**Figure 3:**
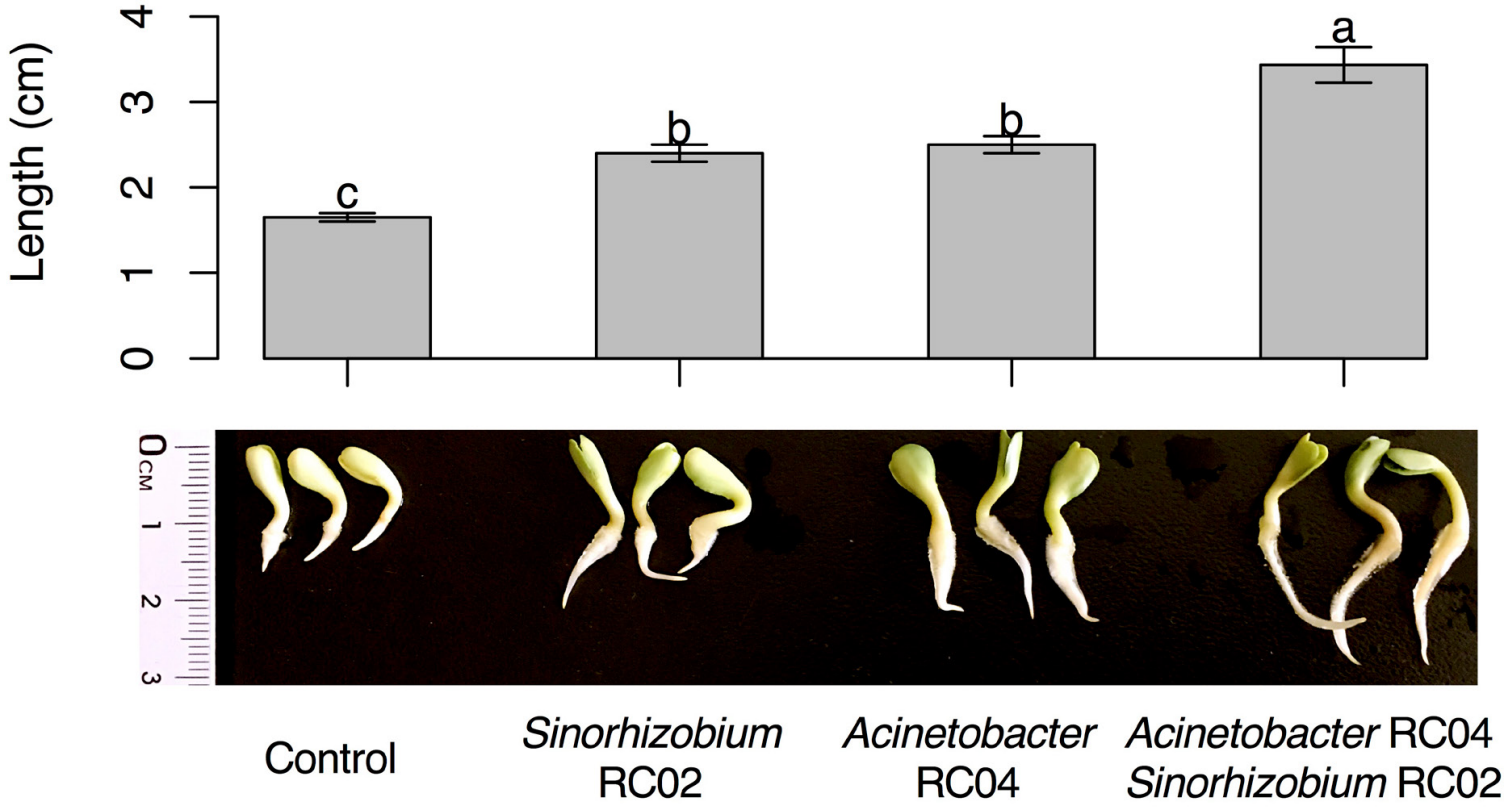
**P**romotion of safflower seed germination by PSB. Bars represent the mean, and error bars show the standard error (*n* = 3). Means marked with different lowercase letters were significantly different (TukeyHSD, *P* < 0.01).

**Figure 4:**
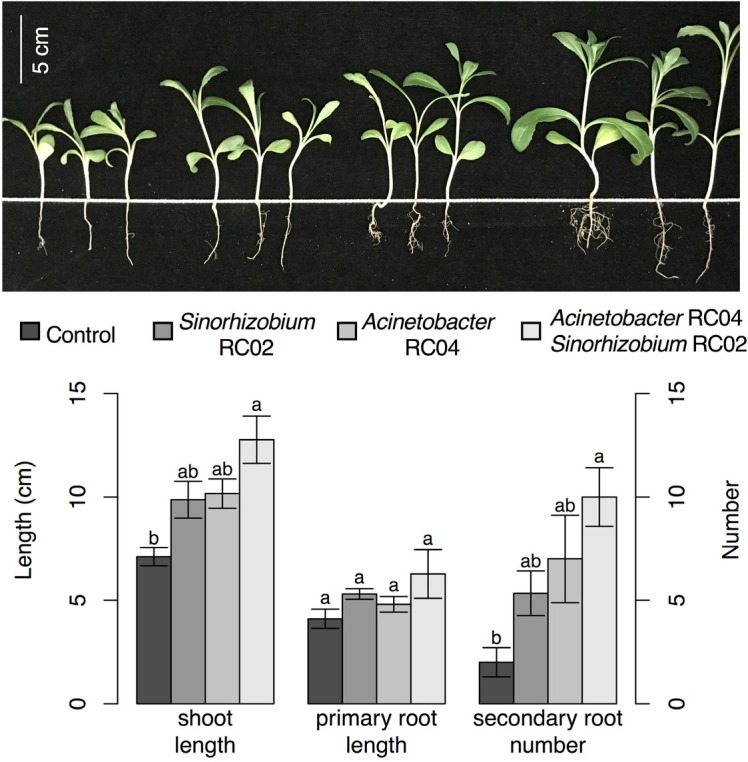
Growth promotion of safflower seedlings by PSB. Bars represent the mean, and error bars show the standard error (*n* = 3). Means within each group marked with different lowercase letters were significantly different (TukeyHSD, *P* < 0.01).

Inoculation of *Acinetobacter* sp. RC04 or *Sinorhizobium* sp. RC02 significantly promoted safflower seed germination (ANOVA, *F*_(3, 8)_ = 97.43, *P* < 10^−5^, Figure 3). In addition, co-inoculation of the two isolates resulted in a significant increase in seedling length compared with single-strain treatment (TukeyHSD, *P* < 10^−4^). Pot trials showed that the co-inoculation had a positive effect on shoot length (ANOVA, *F*_(3, 8)_ = 11.53, *P* = 0.003) and the number of secondary roots (ANOVA, *F*_(3, 8)_ = 8.15, *P* = 0.008) compared with the control (Figure 4).

*Acinetobacter* sp. RC04 and *Sinorhizobium* sp. RC02 showed the ability to promote safflower seed germination and, when co-inoculated, improve seedling growth. These results indicate they might be incorporated into biofertilizers to increase safflower growth [33]. The improvement of plant growth and properties by PSB may be due to several possible mechanisms. PSB alter the plasticity of seeds and roots by changing the soil composition. For example, plant growth-promoting rhizobacteria may improve the solubility of mineral nutrients by releasing organic acids and thereby increasing the vegetative biomass and N and P accumulation in plant tissues, simulating plant growth [34]. This phenomenon, in turn, affects colonization and development of the bacteria [35]. Plant growth-promoting rhizobacteria can induce production of phytoalexin, antibiotics against pathogenic organisms, as well as siderophores, and they colonize root surfaces, thereby out-competing pathogens [34]. Inoculation with plant growth-promoting rhizobacteria can stimulate or inhibit functional community formation and growth in a given symbiotic relationship, depending upon the nature and concentration of secondary metabolites released by the partners in that plant–microbial relationship [34]. Thus, the interactive effect among rhizosphere microorganisms can influence P cycling and promote a sustainable nutrient supply to plants [34]. For instance, inoculation of mixed PSB or co-inoculation with other microorganisms can result in balanced nutrition for plants, such as providing P and N [36]. The present study confirmed the advantage of mixed PSB inoculation. The interactions among PSB, plants and other rhizobacteria create synergistic effects that improve the uptake of individual nutrients [37].

## 4 Conclusions

The present study screened phosphate dissolving bacteria from the rhizosphere soil of safflower. Among these strains, *Acinetobacter* sp. RC04 showed high performance in phosphate solubilization, and the efficiency of the process was influenced by cultivation conditions. *Acinetobacter* sp. RC04 and *Sinorhizobium* sp. RC02 showed the ability to improve safflower seed germination and, when co-inoculated, improve seedling growth. These results indicate the positive role of *Acinetobacter* sp. RC04 and *Sinorhizobium* sp. RC02 in enhancing the biomass of safflower seedlings. Their co-inoculation with plants will be highly beneficial in improving the growth of safflower. Further molecular and biochemical studies of these bacteria will provide efficient ways for incorporating these strains into biofertilizers to promote improved yield of agronomic crops and sustainable agriculture.
