# Supplementary material for: Phosphate-solubilizing Bacteria from Safflower Rhizosphere and their Effect on Seedling Growth
Source: Open Life Sci. 2019 Jul 10;14:246–54. doi: 10.1515/biol-2019-0028 (PMC7874793; doi:10.1515/biol-2019-0028)
Supplement: Supplementary file 1 [file biol-14-246_sm.pdf]

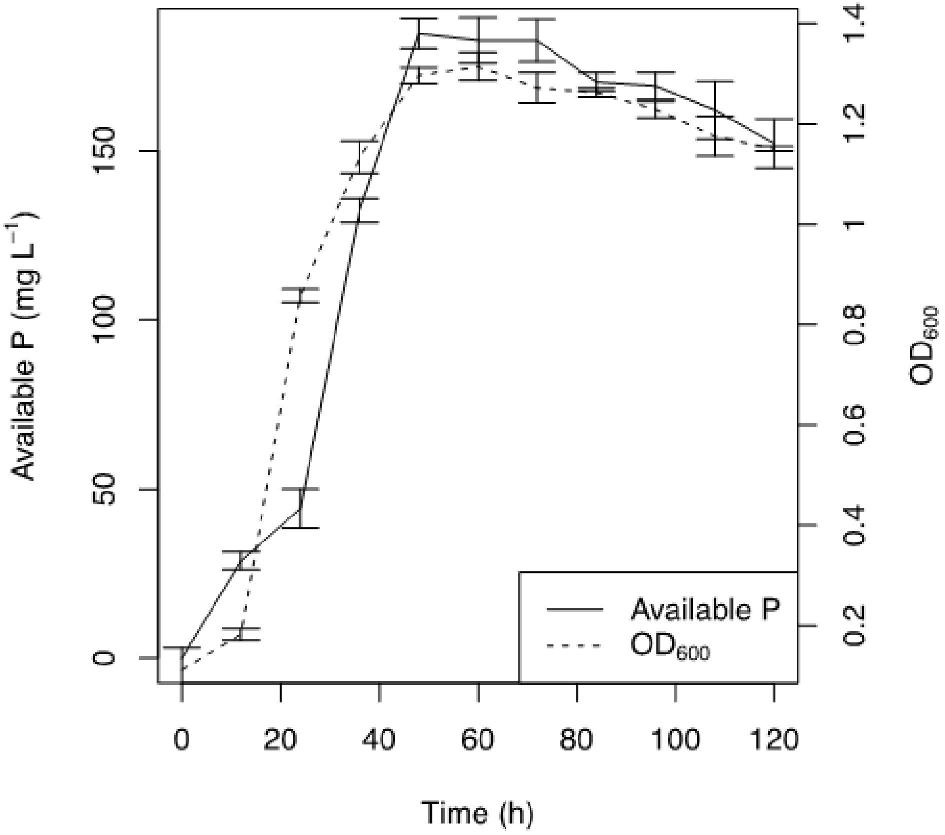

Supplementary figure. Growth profile of Acinetobacter sp. RC04 and culture phosphate solubility. Error bars show standard errors (n = 5). OD_{600} was correlated with P solubilization (Spearman's rank correlation *rho* = 0.95, $P < 10^{-16}$).
